# *QuickStats*: Percentage of Women Who Missed Taking Oral Contraceptive Pills* Among Women Aged 15–44 Years Who Used Oral Contraceptive Pills and Had Sexual Intercourse, Overall and by Age and Number of Pills Missed — National Survey Of Family Growth, United States, 2013–2015^†^

**DOI:** 10.15585/mmwr.mm6636a10

**Published:** 2017-09-15

**Figure:**
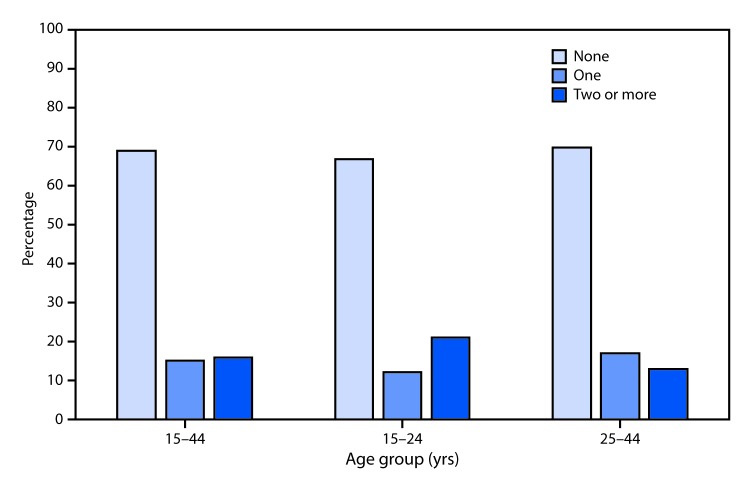
Among women aged 15–44 years who used oral contraceptive pills in the last 4 weeks and had sexual intercourse in the past 12 months, 69% of women reported missing no pills, 15% missed one pill, and 16% missed two or more pills. Across the two age groups (15–24 years and 25–44 years), similar percentages of women aged 15–24 years reported missing no pills (67%) compared with women aged 25–44 years (70%). Similar percentages of women aged 15–24 years reported missing one pill (12%) compared with women aged 25–44 years (17%). A higher percentage of women aged 15–24 years (21%) reported missing two or more pills compared with women aged 25–44 years (13%).

**Source:** National Survey of Family Growth, 2013–2015. https://www.cdc.gov/nchs/nsfg/index.htm.

